# Relationship between herpes simplex virus-1-specific antibody titers and cortical brain damage in Alzheimer’s disease and amnestic mild cognitive impairment

**DOI:** 10.3389/fnagi.2014.00285

**Published:** 2014-10-15

**Authors:** Roberta Mancuso, Francesca Baglio, Simone Agostini, Monia Cabinio Agostini, Maria M. Laganà, Ambra Hernis, Nicolò Margaritella, Franca R. Guerini, Milena Zanzottera, Raffaello Nemni, Mario Clerici

**Affiliations:** ^1^IRCCS, Don C. Gnocchi Foundation – ONLUSMilan, Italy; ^2^Università degli Studi di MilanoMilan, Italy

**Keywords:** HSV-1, Alzheimer’s disease (AD), amnestic mild cognitive impairment (aMCI), magnetic resonance imaging (MRI), voxel based morphometry (VBM), HSV-1 IgG

## Abstract

Alzheimer’s disease (AD) is a multifactorial disease with a still barely understood etiology. Herpes simplex virus 1 (HSV-1) has long been suspected to play a role in the pathogenesis of AD because of its neurotropism, high rate of infection in the general population, and life-long persistence in neuronal cells, particularly in the same brain regions that are usually altered in AD. The goal of this study was to evaluate HSV-1-specific humoral immune responses in patients with a diagnosis of either AD or amnestic mild cognitive impairment (aMCI), and to verify the possible relation between HSV-1-specific antibody (Ab) titers and cortical damage; results were compared to those obtained in a group of healthy controls (HC). HSV-1 serum IgG titers were measured in 225 subjects (83 AD, 68 aMCI, and 74 HC). HSV-specific Ab avidity and cortical gray matter volumes analyzed by magnetic resonance imaging (MRI) were evaluated as well in a subgroup of these individuals (44 AD, 23 aMCI, and 26 HC). Results showed that, whereas HSV-1 seroprevalence and IgG avidity were comparable in the three groups, increased Ab titers (*p* < 0.001) were detected in AD and aMCI compared to HC. Positive significant correlations were detected in AD patients alone between HSV-1 IgG titers and cortical volumes in orbitofrontal (region of interest, ROI1 R_Sp_0.56; *p* = 0.0001) and bilateral temporal cortices (ROI2 R_Sp_0.57; *p* < 0.0001; ROI3 R_Sp_0.48; *p* = 0.001); no correlations could be detected between IgG avidity and MRI parameters. Results herein suggest that a strong HSV-1-specific humoral response could be protective toward AD-associated cortical damage.

## INTRODUCTION

Alzheimer’s disease (AD) is a neurodegenerative disorder involving gray matter (GM) tissue that is now considered to be part of a continuum of clinical and biological phenomena. AD is the most common form of dementia in the elderly, affecting more than 25 million people worldwide, with a prevalence of 5% after 65 years of age, increasing to about 30% in people aged 85 years or older. The amnestic mild cognitive impairment (aMCI) is a syndrome with a high risk of progression to AD, and it could constitute a prodromal stage of this disorder and it represents the borderland condition between normal aging and AD dementia (Petersen, 2004).

Although the pathological lesions observed in AD are well characterized, the causes that trigger the onset of the disease are still unknown. Over the years many environmental and genetic components have been hypothesized to be risk factors for this disease, and a viral component has long been suspected to play a critical role in the pathogenesis of AD (Honjo et al., 2009; Ball et al., 2013). In particular, a number of experimental and epidemiological data suggest that herpes simplex virus type 1 (HSV-1), a neurotropic agent that is often found in elderly brain (Jamieson et al., 1991; Wozniak et al., 2005), could have a key pathogenic role in AD (rev. in Agostini et al., 2014).

After primary infection HSV-1 establishes a long-lasting latent infection in the neurons of the peripheral nervous system (PNS) within the sensory ganglia (trigeminal ganglia). External stimuli including UV light and stress, can induce reactivation of HSV-1 from latency, an event mostly asymptomatic, or resulting in cold sores. Serious neurological complications rarely develop and target the same brain regions that are altered in AD (frontal and temporal cortices as well as the hippocampus).

Although the host immune system plays an important role in reactivating of latent HSV-1, the understanding of immunologic control of HSV-1 in humans remains incomplete.

Recent results stemming from the analysis of the potential role of HSV-1 specific humoral immune responses in neurodegeneration that characterize AD have shown that HSV1-specific antibodies (IgG) titers correlate with cortical GM volume (by voxel-based morphometry, VBM) Thus, analyses performed with VBM in AD patients and healthy controls (HC) indicated the presence of significant correlations between the preservation of temporal and orbitofrontal cortices, and higher HSV-1-specific antibody (Ab) titers (Mancuso et al., 2014). These data suggested a possible protective role for HSV-1-specific humoral immunity in those cerebral regions that are typically affected in AD as well as in HSV-1-associated acute encephalitis.

One of the best parameters to perform a qualitative evaluation of Abs is the measurement of avidity: the relative strength with which Abs bind antigens. In the case of different viral infections it is well known that multiple viral reactivation, generally occurring throughout life, lead to stronger avidity (Thomas et al., 1996; Eisen, 2014). Recent results reported an increased HSV-1 IgG Ab avidity index in aMCI patients compared to AD and HC (Kobayashi et al., 2013), suggesting that viral reactivation is a particularly frequent event in the prodromal stage of AD.

Based on these previous results we investigated whether the correlation we detected between HSV-1-specific Ab titers and magnetic resonance imaging (MRI) VBM parameters could be observed in aMCI as well; possible associations between HSV-1 specific IgG avidity and preservation of brain areas were also analyzed in a subgroup of AD and MCI patients.

## MATERIALS AND METHODS

### SUBJECTS

Eighty-three patients diagnosed with probable AD according to the NINCDS–ADRDA criteria (McKhann et al., 2011), 68 subjects diagnosed with aMCI according to Petersen criteria (Petersen, 2004) and 74 HC were included in the study. All subjects were consecutively recruited at the Fondazione Don Gnocchi, IRCCS in Milano, Italy. AD patients were in mild stage of the disease as determined by both Clinical Dementia Rating (CDR; Morris, 1993) scale (CDR range 0.5–1.5) and mini-mental state examination (MMSE) score (Magni et al., 1996; MMSE mean ± SD 20.0 ± 3.0). To be eligible for the study, aMCI individuals were required to meet the Grundman operational criteria (Grundman et al., 2004): memory complaint, confirmed by an informant; abnormal memory function, documented by previous extensive neuropsychological evaluation; normal general cognitive function, as determined by both CDR scale (Morris, 1993; CDR with at least a 0.5 in the memory domain) and MMSE (Magni et al., 1996) score (MMSE ≥ 24); no impairment in functional activities of daily living as determined by a clinical interview with the patient and informant; no significant cerebral vascular disease (Hachinski score ≤ 4; Rosen et al., 1980); no major psychiatric illnesses with particular attention to exclude subjects with history of depression (Hamilton Depression Rating Scale score ≤ 12; Hamilton, 1960). HC were selected according to the SENIEUR protocol for immune-gerontological studies of European Community’s Control Action Program of Aging (Ligthart et al., 1984) and did not have a family history of dementia or evidence of neurologic disease at the time of enrollment. Demographic and clinical characteristics of the study sample are summarized in **Table 1**. A subgroup of randomly selected patients underwent advanced MRI acquisition (see MRI acquisitions and analyses for details). To increase the diagnostic accuracy analyses of hippocampal volumes, an index of downstream neural injury according to the guidelines for MCI due to Alzheimer’s dementia (Sperling et al., 2011), were also performed in the MRI study sample. The study conformed to the ethical principles of the Helsinki Declaration; all patients or their care-givers gave informed consent according to a protocol approved by the local ethics committee of the Don Gnocchi Foundation.

**Table 1:** Clinical characteristics and serological results of studied subjects.

	*HC*	*aMCI*	*AD*	*Group comparison*
	***N = 74***	***N = 68***	***N = 83***	***p value***
**Clinical characteristics**
Age (years) [median, IQR]	71.5 (63.0–77.0)	75.0 (71.0–80.0)	77.0 (73.0–80.5)	*p* < 0.001
Gender (M:F)	32:42	31:37	33:50	n.s.
MMSE score [mean ± SD]	29.1 ± 1.7	25.5 ± 2.2	20.5 ± 3.0	*p* < 0.001
Level of education (years) [mean ± SD]	8.7 ± 4.2	8.9 ± 3.8	8.2 ± 3.6	n.s.
**Serological results**
HSV-1 seroprevalence (%)	98.6	95.6	97.6	n.s.
HSV-1 IgG titer (AI) [median, IQR]	8.0 (6.0–9.6)	8.8 (7.1–10.5)	9.3 (7.4–10.6)	*p* < 0.001
HSV-1 avidity (%) [median, IQR]*	89.2 (85.0 – 94.6)	91.6 (86.1–96.0)	90.8 (84.6–100.0)	n.s.

AD, Alzheimer’s disease; aMCI, amnestic mild cognitive impairment; HC, healthy control; MMSE, mini mental state evaluation; IQR, interquartile range; SD, standard deviation; HSV-1, herpes simplex virus type 1; AI, antibody index.

*A subgroup of 23 HC, 22 aMCI and 44 AD was considered.

### IMMUNOLOGICAL ANALYSES

Herpes simplex virus 1 serum IgG titers were measured using commercial enzyme immunoassays (BEIA HSV-1 IgG, Technogenetics, Milano, Italy), according the attached protocol.

Briefly, 100 μl of serum samples diluted (1:81) with sample diluent were transferred into the HSV-1 antigen coated polystyrene microwells and the plates were incubated at room temperature (RT) for 30 min. After three washing steps with washing buffer to remove the unbound proteins, 100 μl of horseradish peroxidase conjugate was added to each well and incubated at RT for 30 min. After rewashing step, 100 μl of chromogen/substrate solution were added to each well and incubated at RT for 15 min.

Finally, 100 μl of stop solution were added to each well and the reaction stopped. The wells were read on a plate reader (Labtech International Ltd., UK) and optical densities (ODs) of wells were determined at 450/620 nm. The measured absorbance is proportional to the concentration of HSV-1 IgG antibodies present. HSV-1 Ab levels were expressed as Ab index (AI), calculated by dividing OD measurement generated from the assay by OD cut-off calibrator. Subjects with AI >1.1 were seropositive, whereas subjects with AI < 0.09 were seronegative.

Herpes simplex virus 1 specific IgG avidity was measured with a protein-denaturing agent in a subgroup of seropositive subjects (44 AD, 23 aMCI, and 26 HC); the protocol used for evaluation of avidity is the same above-mentioned, with the addition of 6 M urea to the washing solution at the washing step after plasma reaction. The avidity index (indicated as %) was calculated as follow: anti-HSV-1 Ab titer measured with washing including urea/anti-HSV-1 Ab titer measured with washing without urea.

### ApoE GENOTYPING

Customer-designed Taqman probes for the 112 and 158 codons were used to determine the genotype of apoliprotein E gene (APOE). Primers and probes for the 112 codon are: 112 Forward primer: 5′-GGG CGC GGA CAT GGA G-3′; 112 Reverse primer: 3′-TCC TCG GTG CTC TGG CC-5′; 112 Arg Probe: 5′-CGT GCG CGG CCG-3′-FAM; 112 Cys Probe: 5′-ACG TGT GCG GCC GCC TG-3′-VIC. Primers and probes for the 158 codon are: 158 Forward primer: 5′-TCC GCG ATG CCG ATG-3′; 158 Reverse primer: 3′-GCT CGG CGC CCT CG-5′; 158 Arg probe: 5′-CCT GCA GAA GCG CCT GGC A-3′-FAM; 158 Cys probe: 5′-CCT GCA GAA GGG CCT GGG AGT-3′-VIC.

### MRI ACQUISITIONS AND ANALYSES

#### MRI acquisition protocol

Brain MR images were acquired using a 1,5 T scanner (Siemens Magnetom Avanto, Erlangen, Germany). MR examination was performed in two randomly selected subgroups of AD HSV-1-seropositive patients and aMCI HSV-1 seropositive patients. Demographic and clinical characteristics of the subjects are summarized in **Table 2**. The following sequences were acquired: (1) dual-echo turbo spin echo (TR/TE = 2920/22 ms, FoV = 240 × 180 mm^2^, in-plane resolution = 0.75 mm × 0.75 mm, slice thickness = 4 mm, number of axial slices = 25) and FLAIR sequence (TR/TE = 9000/121 ms, FoV = 240 × 168 mm^2^, in-plane resolution = 0.94 mm × 0.94 mm, slice thickness = 5 mm, number of coronal slices = 24), to exclude patients showing WM hyperintensities outside the normal range; (2) 3-dimensional T1-weighted magnetization prepared rapid gradient echo (MPRAGE; TR/TE = 1900/3.37 ms, FoV = 192 mm × 256 mm, in-plane resolution 1 mm × 1 mm, slice thickness = 1 mm, number of axial slices = 176), to perform VBM analysis and to calculate structural indices.

**Table 2:** Demographical, neuropsychological, immunological, and anatomical information of the MRI subsample.

	*aMCI*	*AD*	*Group comparison*
	***N = 23***	***N = 44***	***p value***
Age, years (median, IQR)	76.0 [69.0–80.75]	77.5 [73–80.5]	n.s.
Gender (M:F)	10:13	17:27	n.s.
Level of education (median, IQR)	8 [5–13]	8 [5–8.5]	n.s.
MMSE total score (median, IQR)	25.7 [24.2–27.1]	20.6 [18.5–23.1]	<0.0001
Nof APOE 𝜀4 carriers	7	22	n.s.
HSV-1 IgG (median, IQR)	8.0 [7.2–9.7]	9.1 [7.3–10.8]	n.s.
HSV-1_IgG Avidity Index, % (median, IQR)	91.6 [86.0–96.2]	90.8 [84.8–99.3]	n.s.
Anatomical characteristics* (cm^**3**^)
L hippocampal volume (mean, SD)	3.08 [0.64]	2.77 [0.48]	0.025
R hippocampal volume (mean, SD)	3.18 [0.58]	2.98 [0.58]	n.s.
GM volume (mean, SD)	662.46 [136.21]	653.24 [34.46]	0.001
cortical GM volume (mean, SD)	540.18 [39.66]	510.66 [30.66]	0.002
WM volume (mean, SD)	676.75 [46.71]	663.59 [33.31]	n.s.
CSF volume (mean, SD)	89.81 [66.36]	85.57 [21.85]	n.s.

aMCI, amnestic mild cognitive impairment; AD, Alzheimer’s disease; MMSE, mini mental state evaluation; HSV-1, herpes simplex virus type 1; GM, gray matter; WM, white matter; CSF, corticospinal fluid; L, left; R, right; IQR, interquartile range; SD, standard deviation.

*One-way analysis of Covariance using the volumetric scaling factor obtained with FSL-SIENAX as covariate.

#### Analysis of T1-weighted structural images

Three different analyses were performed on the T1-weighted structural images in order to provide further information about the underline pathology in this cohort of subjects: (1) computation of hippocampal volumes; (2) VBM analysis; (3) Regression analysis on *a priori* region of interest (ROI).

Computation of hippocampal volumes. Hippocampal volume data were extracted for each subject from high-resolution T1 3D images. Segmentation of right and left hippocampi was performed using FSL’s FIRST method (Patenaude et al., 2011), an approach combining both shape and intensity information within a Bayesian model to segment subcortical structures. After hippocampal segmentation, volumetric data had been obtained in each subject using a specific FSL function.

VBM analysis. The VBM analysis was conducted using VBM8^1^, toolbox of SPM8^2^, running on Matlab 7.6.0^3^. VBM was conducted according to the Unified Method (Ashburner and Friston, 2005). After GM segmentation all images underwent spatial smoothing using a Gaussian kernel (FWHM 8 mm). To identify areas with different GM volume between the two groups direct comparison between AD and MCI patients was performed (two sample *t*-test). Intracranial volume (ICV; obtained by adding up WM volume + GM volume + CSF volume) entered this second level analysis to adjust for potential confounds. Only those areas surviving 0.05 voxel-level FDR corrected threshold have been considered as significant.

**FIGURE 1:**
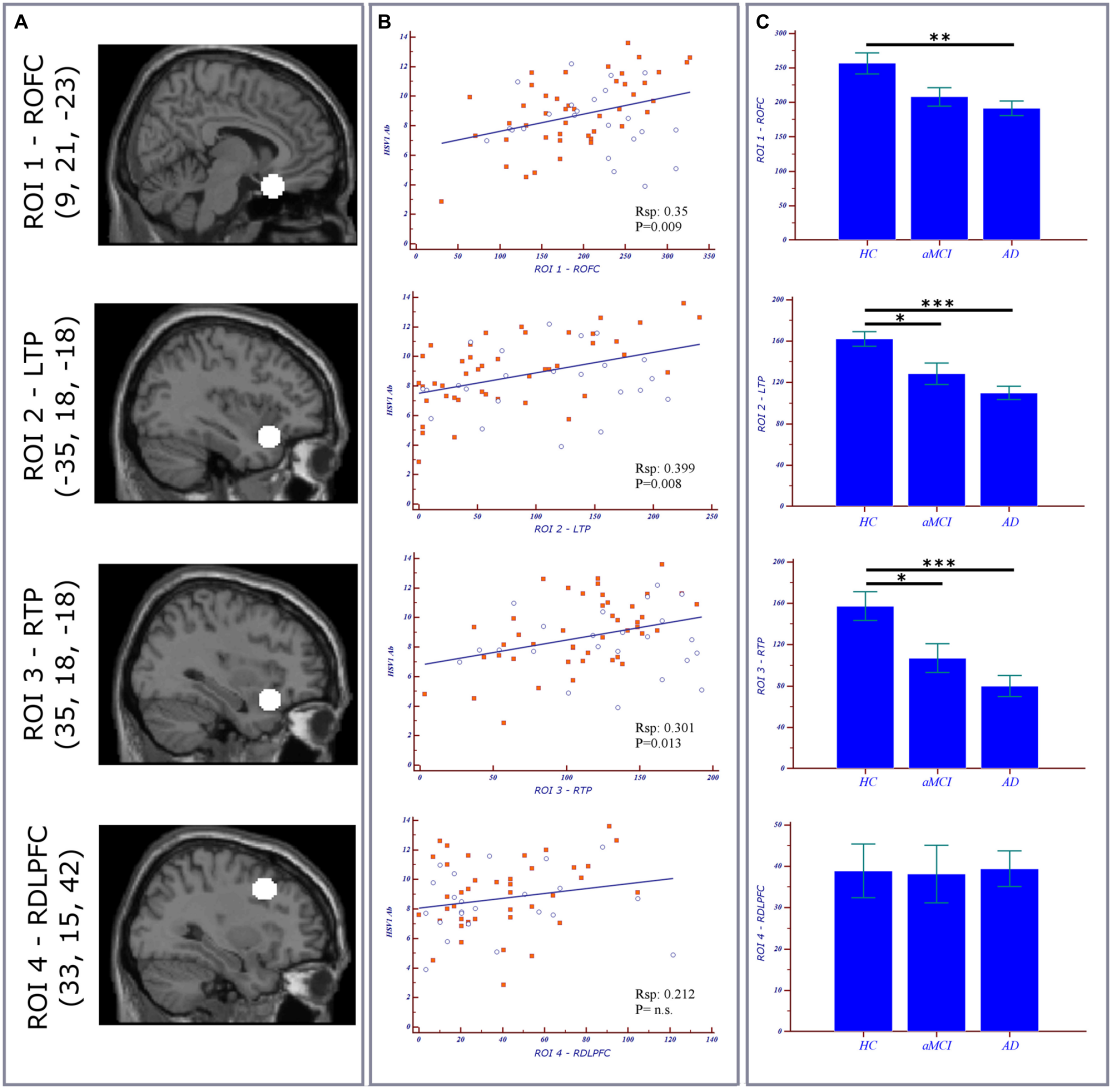
**Region of interest (ROI) Analysis. (A)** ROIs location and relative MNI coordinates (x,y,z). **(B)** Scatterplots representing the correlation between ROIs volumes of both MCI (blue circles) and AD (orange squares) subjects vs. herpes simplex virus 1 (HSV-1)-specific Ab titers. **(C)** Bar charts and error bars representing ROI volumes (mean with SEM) in the three groups. *Post hoc* results are illustrated (*p < 0.05; **p < 0.01; ***p < 0.001). ROFC, right orbitofrontal cortex; LTP, left temporal lobe; RTL, right temporal lobe; RDLPFC, right dorsolateral prefrontal cortex; AD, Alzheimer’s disease; aMCI, amnestic mild cognitive impairment; R_Sp_, Spearman correlation coefficient.

ROI analysis. Normalized and modulated GM volumes of every subject were computed in four 10-mm diameter spherical ROIs. Three ROIs’ centers were located as the significant peak obtained with the voxel-wise correlation of GM volumes and HSV-1-specific Ab titers in AD patients (Mancuso et al., 2014): ROI1 in orbitofrontal cortex, ROI2 in left inferior frontal gyrus/temporal pole, and ROI3 right inferior frontal gyrus/temporal pole. One additional control ROI4 was positioned in dorsolateral prefrontal cortex, an area which is preserved by the HSV-1 and which is affected in moderate to severe AD pathology. The ROIs and the relative MNI coordinates were illustrated in **Figure 1A**. The ROI-volumes were computed after that a threshold of 0.75 was applied to the segmented GM map, in order to increase the certainty of belonging to GM. Statistical linear regression analyses were performed between GM values in the ROIs and HSV-1 Ab levels and IgG anti-HSV-1 antibodies avidity.

### STATISTICAL ANALYSIS

The statistical analyses were accomplished using commercial software (SPSS for Windows, V 18.0; SPSS Inc). We compared aMCI, AD, and HC on demographic data, using the Chi-square test and One-way ANOVA with Bonferroni *post hoc* test for categorical and continuous variables, respectively.

Differences in immunological data (HSV-1 Ab levels and IgG anti- HSV-1 antibodies avidity) among groups where tested using One-way ANOVA with Bonferroni *post hoc* test after excluding the presence of any significant covariate.

In a randomly selected subgroup of patients (AD, aMCI), RM-ROI data were collected and differences between groups were evaluated by means of Mann Whitney U, Chi-square test and One-way analysis of covariance using the volumetric scaling (V-Scaling) factor obtained with FSL-SIENAX as covariate, in order to take into account the differences in head size among subjects. Following our previous work, GM values from ROIs of both MCI and AD were correlated with immunological data by means of Spearman’s correlation coefficient.

All the quantitative variables were described using mean and SD or median and interquartile range (IQR) and an alpha = 0.05 was considered significant.

## RESULTS

### DEMOGRAPHICAL AND ANATOMICAL CHARACTERISTICS OF THE PARTICIPANTS

Gender and educational levels were similar among the three groups (HC, aMCI, and AD). Age differed among groups (*p* < 0.001; HC < AD and HC < aMCI *p*_corr_ < 0.05; AD vs. aMCI n.s.), thus this parameter was considered as covariate in all the statistical analyses. Global cognitive levels (MMSE) were, as per definition, reduced in AD and aMCI compared to HC (*p* < 0.0001) and lower in AD than aMCI (*p* < 0.0001; **Table 1**).

The subgroup of aMCI individuals and AD patients in whom MRI analyses were performed were comparable for age, gender, years of education, and proportion of APOE 𝜀-4 carriers. Again, a significant difference was found for MMSE values, accordingly with the adopted inclusion criteria (**Table 2**).

### HSV-1 SEROPREVALENCE, TITERS, AND AVIDITY

**FIGURE 2:**
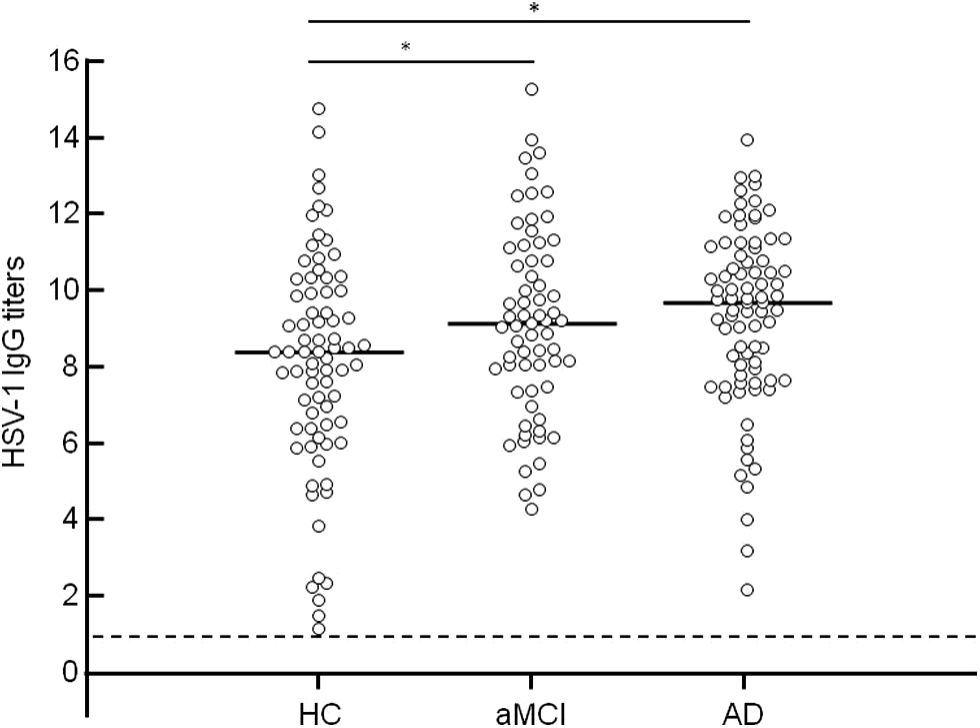
**Anti HSV-1 antibody (Ab) titers in healthy control, aMCI, and AD groups.** Horizontal lines represent the median value. Dotted line represents threshold value of HSV-1 seropositivity. Statistical significance was evaluated using One-way ANOVA with Bonferroni correction for multiple comparisons (significance level: p_corr_ < 0.05). *Significant compared to aMCI and AD group vs. HC.

Although no differences were observed among the groups when HSV-1 seroprevalence was analyzed (AD = 97.6%; aMCI = 95.6%; HC = 98.6%), ANOVA comparisons showed that the three groups significantly differed with regard to HSV-1 Ab titers (*p* < 0.001). Thus, significantly higher HSV-1 IgG titers were detected in AD (median: 9.3; range: 7.4–10.6 AI) and aMCI (median: 8.8 AI; range: 7.1–10.5 AI) individuals compared to HC (median: 7.9 AI; range: 6.0–9.6 AI; HC vs. AD *p* = 0.0049; HC vs. aMCI *p* = 0.025; **Figure 2**).

No differences were found among the three groups when HSV-1 IgG avidity indexes were analyzed. Thus, median HSV-1 avidity for AD patients was 90.8% (IQR, 84.6–100.0), 91.6% (86.1–96.0) for MCI patients, and 89.2% for HC (85.6–94.6).

As expected by the limited range of MMSE score no correlations were detected between MMSE level and HSV-1 IgG titers or avidity indices. No correlations were obtained between age, APOE4 status and the same indices neither in the overall group nor in the groups considered separately.

### MRI RESULTS AND CORRELATIONS

Structural MRI confirmed the pattern of GM atrophy typical of aMCI and AD. In particular, left hippocampal volumes were significantly different across groups (*p* < 0.001), and larger in HC compared both to MCI and AD (*p* < 0.001 and *p* = 0.02, respectively), and in MCI compared to AD (*p* < 0.05), as shown by *post hoc* tests with Bonferroni correction. Right hippocampal volumes were significantly different across groups as well (*p* < 0.001), and were larger in HC compared both to MCI and AD (*p* < 0.001 and *p* = 0.02, respectively) as shown by *post hoc* tests with Bonferroni correction. No statistically significant differences were found between MCI and AD.

**Table 3** shows the results of the VBM on structural MRI data and reveals a well-known pattern of GM atrophy in AD patients compared to MCI patients: patients with AD were significantly more atrophic than aMCI subjects in several brain regions including bilateral hippocampus and parahippocampal gyrus, right middle temporal gyrus (right>left), right fusiform gyrus, and right amygdala. AD patients also showed decreased GM volume compared to aMCI subjects bilaterally in cerebellum and posterior cingulate cortex.

**Table 3:** VBM results.

MNI coordinates			Size	Peak intensity	Cortical area
*x*	*y*	*z*		*z value*	aMCI > AD*
25.5	-57	-3	129	3.07	R Parahippocampal gy/Limbic lobe
33	-19.5	-31.5	604	3.49	R Parahippocampal gy
40.5	-73.5	3	10395	4.50	R middle temporal gy
43.5	-42	-27	364	3.25	R fusiform gy
19.5	-13.5	-10.5	335	3.79	R amigdala
-22.5	22.5	-16.5	4787	4.55	L inf frontal gy/temporal pole
-52.5	-61.5	24	9975	5.18	L mid temporal gy
-18	-10.5	-9	378	3.71	L hippocampus/parahippocampus
-16.5	-27	43.5	520	3.62	L post cingulum
15	-31.5	45	184	3.23	R post cingulum
19.5	-63	48	2027	4.68	R precuneus
24	-43.5	-55.5	563	4.02	R cerebellum
-36	-81	-37.5	1381	3.88	L cerebellum

AD, Alzheimer’s disease; aMCI, amnestic mild cognitive impairment; L, left; R, Right; gy, gyrus; mid, middle; inf, inferior; post, posterior.

*Brain areas surviving 0.05 voxel-level FDR corrected threshold with an extend cluster of 100 voxels.

Correlations between GM values from ROIs of both MCI and AD and HSV-1-specific Ab titers are shown in **Figure 1B**. To summarize: a statistically significant positive correlation was detected between AD cortical atrophy in orbitofrontal (ROI1 R_Sp_0.56; p = 0.0001) and bilateral temporal cortices (ROI2 R_Sp_0.57; p < 0.0001; ROI3 R_Sp_0.48; p = 0.001) and HSV-1 IgG titers. Such correlation was not observed in aMCI although areas of increased atrophy compared with HC were seen in the bilateral temporal cortex (**Figure 1C**). No correlations were obtained between the dorsolateral prefrontal cortex (ROI4), an area which is preserved in aMCI and in mild AD, and HSV-1-specific Ab titers either. Finally, the statistical linear regression analyses performed between GM values in the ROIs and HSV-1 avidity index did not show any statistically significant differences between aMCI and AD.

## DISCUSSION

A pathogenic role for HSV-1, a neurotropic agent, in AD has long been suspected (Jamieson et al., 1991; Ball et al., 2013; Agostini et al., 2014). HSV-1 induces inflammation in the brain areas that are mostly affected by AD (Damasio and Van Hoesen, 1985; Kennedy and Chaudhuri, 2002; Sokolov and Reincke, 2012), and it contributes to senile plaque formation (Wozniak et al., 2007). In a previous study we found a correlation between cortical atrophy in those areas of the brain that are strongly related to pathology in AD patients and titers of HSV-1-specific Ab, suggesting that HSV-1-humoral immunity could be a protective factor in AD (Mancuso et al., 2014).

Aim of the present study was to further investigate the link between neurodegeneration and HSV-1-humoral immunity by expanding our analyses to aMCI patients. aMCI is defined as a mild cognitive deficit in the memory domain in the absence of dementia; this condition was proposed to fill the gap between normal and dementia-type pathological aging, assuming the existence of a cognitive continuum between normal aging and AD, the main cause of dementia (Petersen, 2004).

As expected, and confirming previous data, HSV-1 seroprevalence did not differ when AD or aMCI patients were compared to healthy controls (Mancuso et al., 2014). IgG Ab titers, however, were significantly increased in AD compared to HC, reinforcing the borderline value of significance found when a smaller group of individuals was analyzed (Mancuso et al., 2014). An additional significant result obtained in the present study is that HSV-1 IgG titers were significantly increased in aMCI subjects as well. These results are novel and suggest that this phenomenon occurs already in the early phase of the cognitive impairment decline, before the diagnosis of AD, and that a more robust immune response may be elicited by HSV-1 infection in AD and aMCI subjects.

These data also support the hypothesis that the alterations of the blood–brain barrier (BBB), a mechanism that might interact and facilitate the effect of neurodegeneration (Carvey et al., 2009; De Vries et al., 2012; Burgmans et al., 2013), occurs before the clinical symptoms (Bäckman et al., 2005). The selective involvement of bilateral medial temporal structures in aMCI shown with MRI results supports this assumption. In agreement with several previous publications, the results of MRI structural data showed that the aMCI group is midway between HC and AD patients on the typical AD pattern of distribution of neurodegeneration which starts from medial-temporal regions, then spreads to medial-parietal cortex and orbitofrontal regions, and finally to other neocortical association areas (Morbelli et al., 2010; Gili et al., 2011).

Results herein also show a lack of correlation between AD cortical volumes in prefrontal cortex and HSV-1 humoral immunity and further confirm the correlation between AD cortical atrophy in temporal and orbitofrontal cortices and HSV-1 humoral immunity (Mancuso et al., 2014).

Given the association between brain atrophy and the increased BBB permeability in temporal lobe of AD patients (Matsumoto et al., 2007), it is tempting to hypothesize that concentration of HSV-1-specific Ab in the CNS would result in down-regulation of HSV-1 activity in those brain regions where BBB permeability is augmented. To confirm this hypothesis it will be necessary to precisely quantify the actual titers of Ab that enter the brain when the BBB permeability is altered.

Interestingly, such a correlation was not observed in aMCI, even if area of increased atrophy in the temporal cortex was seen in these individuals. The small number of aMCI analyzed as well as the fact that not all aMCI will evolve into AD may play a role in such findings. In fact, several studies reported that not all individuals with MCI (amnestic or non-amnestic, including those with positive biomarkers) progress to AD, particularly in community-based settings, and the typical rates at which aMCI patients progress to AD is 14–18% per year (Petersen et al., 2009; Ganguli et al., 2011; Brodaty et al., 2013). Future studies with larger samples of aMCI patients, as well as a longitudinal evaluation of the present sample will help in the comprehension of these results.

Although several evidences indicate that HSV-1 plays a key role in the disease (e.g., the HSV-1 specific localization in amyloid plaques – Wozniak et al., 2009), it cannot be excluded that a robust immune response may have a protective role against AD development making HSV-1 suppression simply a secondary effect. An alternative explanation could be that high Ab levels reflect viral-induced host responses due to an attempt to control HSV-1 in those affected brain regions showing an increased antigen presentation.

To further study the relationship between HSV-1 infection and neurodegeneration, HSV-1 avidity was also investigated in the current study. Recently Kobayashi et al. (2013) reported that the avidity of HSV-1-specific IgG is increased in aMCI patients compared to HC and AD subjects, suggesting that an abnormal rate of HSV-1 reactivation can contribute to the neurodegeneration process in the initial stage of dementia. Our results did not evidence differences in the avidity of these Ab in the three groups analyzed. Higher avidity is considered to be a marker of recent primary infection (Odievre et al., 2002) and many studies have shown its utility in the evaluation of immune status during pregnancy, (Fitzgerald et al., 1988; De Ory et al., 1993; Junker and Tilley, 1994; Boppana and Britt, 1995), as this parameter can recognize acute infections that are associated with a high neonatal risk. Data herein, thus, probably reflect that primary HSV-1 infection occurred early in life; if that is the case, clear differences about this index are not appreciable, as already attested by Hashido et al. (1997).

In conclusion, our analyses confirm the hypothesis that the presence of potent HSV-1-specific immune responses plays a protective role against AD-associated GM degeneration, supporting the idea that HSV-1 might indeed play a critical role in the pathogenesis of this disease.

## Conflict of Interest Statement

The authors declare that the research was conducted in the absence of any commercial or financial relationships that could be construed as a potential conflict of interest.

## Footnotes

1 http://dbm.neuro.uni-jena.de/vbm/

2 www.fil.ion.ucl.ac.uk/spm/

3 www.mathworks.it/products/matlab
